# Are COVID-19 survivors at increased risk for suicide?

**DOI:** 10.1017/neu.2020.21

**Published:** 2020-05-04

**Authors:** Leo Sher

**Affiliations:** James J. Peters Veterans’ Administration Medical Center, Bronx, NY, USA; Department of Psychiatry, Icahn School of Medicine at Mount Sinai, New York, NY, USA

The coronavirus disease 19 (COVID-19) outbreak first emerged in China late last year. The COVID-19 epidemic has spread to all continents. Millions of people got sick with COVID-19. In this letter to the editor, I suggest that the COVID-19 survivors especially individuals who had severe COVID-19 are at increased suicide risk. Suicidality among individuals who had COVID-19 may be related to both psychological and neurobiological factors.

Psychological factors that may increase suicide risk among COVID-19 patients include learning about their diagnosis, anxiety, and distress related to symptoms of the disease and stress related to hospitalisation and hospital treatment. Realising that they have COVID-19 may be very stressful, especially for low-resilient individuals. Symptoms of the disease, especially severe symptoms, as well as social isolation and fear of infecting other people may lead to a serious psychological trauma. Individuals who needed an admission to an intensive care unit (ICU) are at especially high risk of developing post-traumatic stress disorder (PTSD), depression, anxiety, sleep abnormalities, and cognitive impairments (McGiffin *et al.*, 2016). Ventilation adjustments, gaps in anaesthesia and/or analgesia, long sedation, restraint use, and other ICU-related factors contribute to a profound psychological effect of ICU hospitalisations. A recent study in China indicated that 96.2% of recovering COVID-19 patients had significant post-traumatic stress symptoms (Bo *et al.*, 2020).

Multiple lines of evidence indicate that stress-related disorders including depression, PTSD, and sleep disorders are associated with suicidal ideation, suicide attempts, and death by suicide (Sher, 2019). Sleep abnormalities are a stand-alone risk factor for suicidal behaviour. Cognitive impairments are also associated with suicidality (Sher, 2019). Many recovering COVID-19 patients have physical symptoms for a long time and experience psychosocial difficulties such as loss of employment and financial issues. Both physical symptoms and psychosocial stressors contribute to suicidal behaviour (Sher, 2019).

COVID-19 has neurobiological effects (Asadi-Pooya & Simani, 2020). Studies have shown human coronavirus infections are associated with neuroinvasion and neurotropism (Asadi-Pooya & Simani, 2020). A recent review of the effect of COVID-19 on the central nervous system indicates that neurological manifestations are present in about 25% of the patients (Asadi-Pooya & Simani, 2020). Headache, dizziness, acute ischaemic stroke, ataxia, seizures, and other neurological conditions have been observed in COVID-19 patients. Neurological conditions including ischaemic stroke and headache are associated with increased suicide risk (Hudzik & Marek, 2014).

COVID-19 survivors should be regarded as individuals at elevated risk for suicide. The single most significant predictor of suicide is the presence of depression. Recovered COVID-19 patients need to be screened for depression and suicidality. Many coronavirus disease survivors will need long-term psychological interventions. There should be specific strategies to enhance the psychological condition of COVID-19 survivors and reduce suicidality in this population. We need to examine what kind of early interventions in coronavirus disease survivors may decrease psychiatric morbidity and suicidality in the future.
